# Efficacy and safety of calcineurin inhibitor therapy in lupus nephritis: a systematic review and network meta-analysis

**DOI:** 10.3389/fimmu.2025.1670134

**Published:** 2026-01-02

**Authors:** Yan Wu, Wenting Cai, Yao Yao, Jinping Zhang

**Affiliations:** 1 Department of Pharmacy, Nanjing Drum Tower Hospital, School of Basic Medicine and Clinical Pharmacy, China Pharmaceutical University, Nanjing, Jiangsu, China; 2 Department of Pharmacy, Nanjing Drum Tower Hospital, Affiliated Hospital of Medical School, Nanjing University, Nanjing, Jiangsu, China

**Keywords:** calcineurin inhibitor, efficacy, lupus nephritis, network meta-analysis, safety

## Abstract

**Introduction:**

The aim of this study is to compare the efficacy and safety of calcineurin inhibitors in the treatment of lupus nephritis.

**Methods:**

We systematically searched several electronic databases (PubMed, Embase, Cochrane Library, and Web of Science) and the clinical trial registry ClinicalTrials.gov from their inception to March 1, 2025. Our study included randomized controlled trials that enrolled adult lupus nephritis patients receiving calcineurin inhibitors. The outcomes included renal remission rate and incidence of adverse reactions. The network meta-analysis was performed using Stata 14.0.

**Results:**

Sixteen randomized controlled trials (with a total of 1994 patients) met the inclusion criteria. Regarding total remission, voclosporin-based triple therapy (mycophenolate mofetil + steroid) ranked highest (surface under the cumulative ranking curve [SUCRA] = 84.3%), followed by tacrolimus-based triple therapy (SUCRA = 78.0%.). For safety outcomes, no statistically significant intergroup differences were observed in adverse events, serious adverse events, or adverse events leading to treatment discontinuation. However, further analysis revealed that voclosporin-based therapy was associated with the highest infection rate (SUCRA = 20.6%), indicating a potential safety concern compared to other regimens; tacrolimus-based therapy had the second-highest infection risk (SUCRA = 27.0%).

**Conclusions:**

Voclosporin- and tacrolimus-based triple therapies (both combined with mycophenolate mofetil and steroid) demonstrated high efficacy in lupus nephritis patients. Based on indirect comparisons, the voclosporin regimen appeared to be the most effective. However, it also posed a significantly higher infection risk than other treatments. This underscores that while pursuing high remission rates, vigilant infection monitoring and proactive management are imperative.

**Systematic Review Registration:**

https://www.crd.york.ac.uk/prospero, identifier CRD420251084364.

## 1 Introduction

In systemic lupus erythematosus (SLE), lupus nephritis (LN) represents a severe renal involvement marked by proteinuria, hematuria, and impaired kidney function (1, 2). Among SLE patients, 40%–60% develop LN, especially when kidney involvement occurs. Within the first 10 years following the initial diagnosis of SLE, 5–20% of patients with lupus nephritis (LN) progress to end-stage renal disease (ESRD) (3). While immunosuppressive regimens are essential for controlling disease activity, their distinct toxicity profiles—manifested as heightened risks of infection, osteoporosis development, and adverse effects on cardiovascular and reproductive systems—remain persistent challenges in clinical management (3–5). If left untreated or improperly managed, SLE patients’ renal function will deteriorate (6), resulting in higher morbidity and mortality rates (7, 8). Therefore, the main treatment goal for LN is to control disease activity and prevent relapse and progression.

Therapeutic protocols for LN typically involve initial treatment with mycophenolate mofetil (MMF) or cyclophosphamide (CTX) in combination with steroid as primary intervention. For long-term disease management, maintenance therapy generally consists of either MMF or azathioprine (AZA) administered concomitantly with low-dose steroid regimens (9–11). With 1-year efficacy rates spanning 30.4% to 66.2%, these treatment approaches show that beneficial renal responses are associated with better prognostic outcomes (12). Despite demonstrating therapeutic efficacy, this treatment modality presents significant clinical challenges due to associated adverse events, particularly myelosuppression, infectious complications, ovarian failure, and gastrointestinal disturbances. Approximately 10% of lupus nephritis patients progress to end-stage renal disease following long-term treatment, indicating poor clinical prognosis (13). Therefore, additional treatment regimens are needed to improve the prognosis of LN.

Calcineurin inhibitors (CNIs), such as Cyclosporine A (CsA), Tacrolimus (TAC), and the newer Voclosporin (VOC), serve as powerful immunosuppressants commonly employed in LN treatment. These agents exert their effects primarily by blocking the nuclear factor of activated T cells (NFAT) transcription factor family, which leads to reduced effector T-cell activity (14). Additionally, CNIs also suppress transcription of interleukin-2 (IL-2) early activation genes and curtail T cell-mediated generation of proinflammatory cytokines including tumor necrosis factor-α (TNF-α), interleukin-1β(IL-1β), and interleukin-6(IL-6) (15). Beyond immunosuppression, CNIs may directly stabilize podocyte actin cytoskeleton, mitigating proteinuria—a feature distinguishing them from MMF/CTX (16, 17).

In clinical practice, CNIs are generally recommended for moderate-to-severe LN or cases demonstrating poor response to initial therapies like MMF or CTX. To optimize therapeutic outcomes while minimizing toxicity, CNIs are typically combined with low-dose glucocorticoids. Emerging evidence suggests that adding CNIs to standard-of-care induction therapy may significantly improve complete renal remission(CR) in LN patients (18). VOC, an advanced CNI, features refined pharmacokinetics with more stable blood concentrations and a lower risk of drug accumulation compared to CsA or TAC (19), has shown promising efficacy in recent phase III trials (20).

Although previous studies have employed network meta-analyses to compare the safety and efficacy of different CNIs (21, 22), these prior meta-analyses either included a limited number of studies or failed to incorporate the VOC treatment regimen. Hence, the main aim of this research was to conduct a network meta-analysis (NMA) comparing the safety and efficacy of different treatment regimens for CNIs in patients with LN.

## 2 Methods

### 2.1 Protocol and registration

The study protocol was registered with PROSPERO (registration number: CRD420251084364) and the methodology of the PRISMA guidelines was followed.

### 2.2 Search strategy

We performed a comprehensive systematic search across four major databases (PubMed, EMBASE, Cochrane Library and Web of Science) and the clinical trial registry ClinicalTrials.gov from their inception through March 1, 2025. We included randomized controlled trials (RCTs) evaluating the use of CNIs in LN patients across all age groups. The search terms incorporated key terms including “lupus nephritis”, “voclosporin”, “cyclosporine A”, and “tacrolimus”, with results restricted to RCTs. Duplicate literature was removed using Endnote 21.4. After removing duplicate records using EndNote software, we assessed studies based on two primary endpoints: renal remission and adverse event profiles.

### 2.3 Inclusion and exclusion criteria

The inclusion criteria were as follows: (1) Patients with LN regardless of age or sex; (2) In the experimental group, patients received CNIs (VOC, TAC or CsA). In the control group, patients received non-CNI regimens (corticosteroids alone or combined with CTX, MMF or AZA); (3) Outcome measures: the primary outcome indicators of effectiveness included the number of patients in CR or the number of patients in total renal remission (TR). The safety indicators mainly included adverse events (AE), serious adverse events (SAE), AE leading to discontinuation and infection events; (4) Study design: Only RCTs were included; (5) Language type: No restrictions.

Exclusion criteria: (1) Research such as review, abstract, report, editorials, and animal experiments; (2) Studies with incomplete outcome data; (3) Repeated published research; (4) non-RCT designs.

### 2.4 Literature selection and data collection

Two investigators independently screened the literature based on predefined eligibility criteria. After reviewing titles, abstracts, and full texts, irrelevant studies were excluded. Data extraction utilized Excel 2019. The following data were independently collected by both investigators: the extracted content includes the name of the first author, the year of publication, study region, the number of patients, intervention measure, drug dosage, treatment duration, diagnosis of LN and NCT number. Any discrepancies were adjudicated by a third reviewer through consensus discussions.

### 2.5 Outcomes

The effectiveness outcomes included rates of CR, partial renal remission (PR), and TR. CR is commonly defined as proteinuria reduction to ≤ 0.5 g/day with concurrent stabilization or recovery of renal function. In contrast, PR, though definitions differ across trials, typically requires a clinically meaningful reduction in proteinuria (e.g., > 50% decrease from baseline) without achieving CR thresholds, alongside preserved or improved kidney function. The definition of TR is the sum of CR and PR.

Safety outcomes included the incidence of AE, SAE, AE leading to treatment discontinuation, and infections. SAE were defined as events occurring during clinical trials that met any of the following criteria: necessitating hospitalization, prolonging existing hospitalization, causing persistent disability, resulting in life-threatening conditions, or death.

### 2.6 Quality assessment

We evaluated the bias risk of the included literature according to the RCT bias assessment tool in Cochrane’s manual. The bias risk assessment comprehensively considered seven aspects, including random sequence generation, allocation concealment, blinding of participants and personnel, blinding of outcome assessment, incomplete outcome data, selective reporting, and other bias. Each criterion was categorized as presenting a low risk, high risk, or ambiguous bias risk. Ambiguous bias risk indicates a lack of information or uncertainty regarding potential bias. The assessment of quality was conducted independently by two authors, and disagreements were resolved through intergroup discussion.

### 2.7 Statistical analysis

The risk of bias and heterogeneity of the included studies were assessed using Review Manager (RevMan) 5.4. To assess heterogeneity, the I² statistic was employed. Subsequently, a frequentist random-effects NMA was performed using Stata (version 14.0) with the “network” and “mvmeta” packages. The analysis included the generation of network evidence plots, calculation of the Surface Under the Cumulative Ranking Curve (SUCRA), league plots, and funnel plots. Risk ratio (RR) and 95% confidence interval (95%CI) were used as effect sizes to analyze the statistics. Global inconsistency tests were performed; a *P* value > 0.05 indicated no significant inconsistency. Local inconsistency was evaluated using node-splitting analyses by comparing direct and indirect effect estimates for each intervention contrast, with a p-value < 0.05 defined as statistically significant. SUCRA values were calculated to rank the treatment regimens for each outcome. Higher SUCRA values (closer to 100%) indicate a higher probability of the regimen being the best for that outcome (23). A sensitivity analysis was conducted to ensure the robustness of the overall results. The publication bias was evaluated by using the funnel plot. An assessment of publication bias was performed through both a visual inspection of the funnel plot and the statistical application of Egger’s regression. A P-value of < 0.05 was considered indicative of statistically significant publication bias. Finally, a cluster rank plot was constructed based on the SUCRA values of the treatments to simultaneously display their ranking across both benefit and risk outcomes.

## 3 Results

### 3.1 Description of included studies

**Figure 1:**
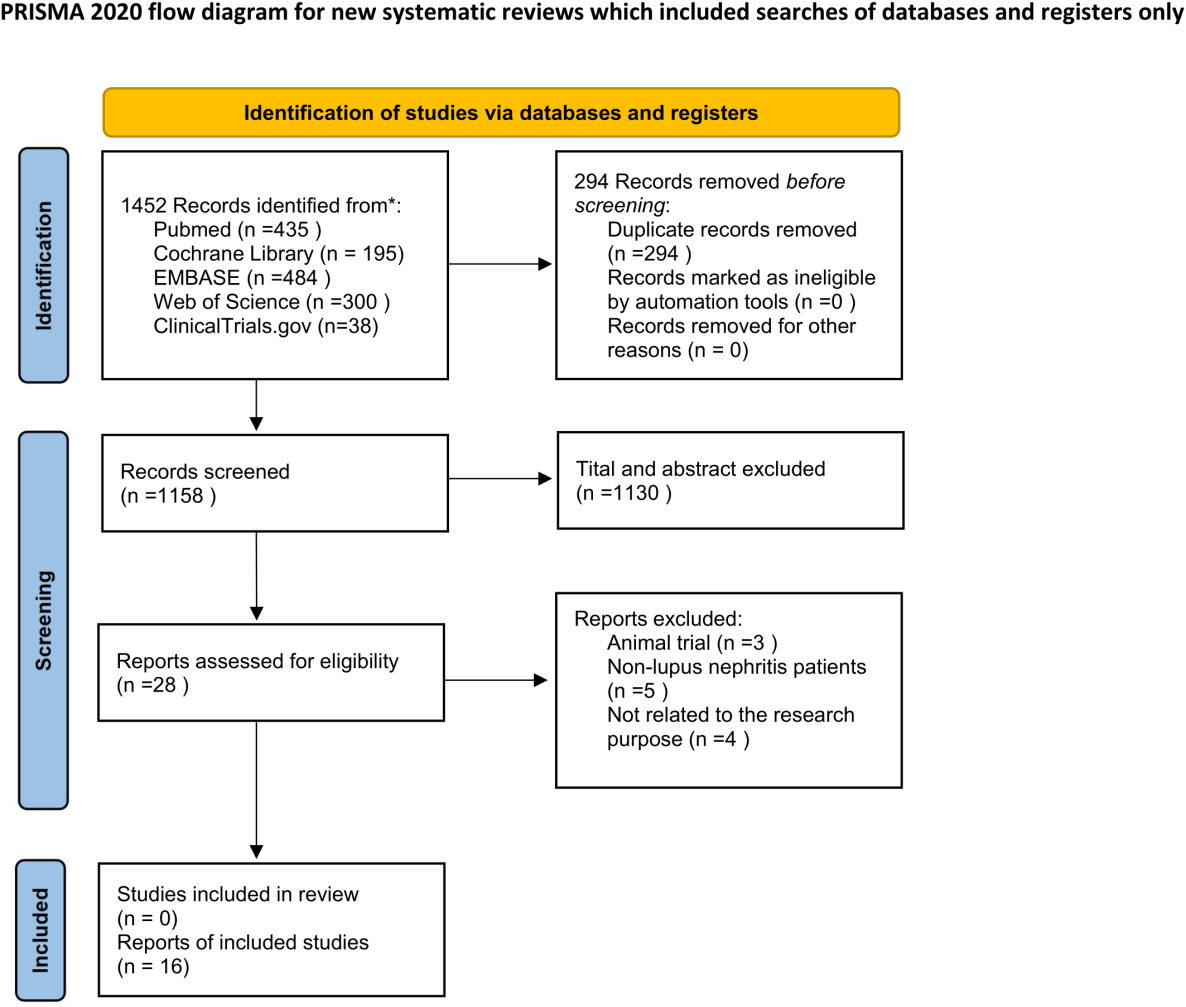
Flow diagram of the selection process of studies.

Initially, 1452 studies were considered for this review. After removing 294 duplicates and reviewing the titles, keywords, and abstracts, 28 articles were selected for full-text evaluation. Out of these, 12 articles did not meet the criteria and were excluded, resulting in a final selection of 16 RCTs (20, 24–38). The literature retrieval process was shown in **Figure 1**. These RCTs included twelve two-armed trials and four three-armed trials. The study population comprised 1994 patients, with eleven studies being multicenter trials and five being single-center trials. The follow-up duration ranged from 24 weeks to 96 weeks. VOC was investigated in two studies, whereas TAC and CsA were examined in nine and five studies, respectively. The validity of the NMA relies on the transitivity assumption, which requires that the included trials be comparable in key clinical and methodological characteristics. To evaluate this, we systematically compared effect modifiers across the trials (details provided in **Supplementary Table S1**). The results indicated significant clinical homogeneity among the patient populations: the majority were Asian with active proliferative lupus nephritis (Class III/IV); baseline disease severity indicators, such as proteinuria and estimated glomerular filtration rate (eGFR), fell within narrow and clinically comparable ranges; and induction steroid regimens were largely consistent. These findings support the plausibility of the transitivity assumption. **Table 1** summarizes the basic identification information and intervention schemes of the included studies.

**Table 1:** Study characteristics of included RCTs.

Author, year	Study region	Median/mean age (years)	female	NCT number	N	Experimental group	Dose	Control group	Treatment duration (wk)
Rovin BH, 2021 (20)	Worldwide	31.5	87.40%	NCT03021499	357	VOC+MMF+prednisone	23.7 mg bid	MMF+prednisone	52
Rovin BH, 2018 (34)	Worldwide	31.7	86.80%	NCT02141672	265	VOC+MMF+prednisone	23.7/39.5 mg bid	MMF+prednisone	48
Liu Z, 2015 (29)	China	31.9	90.90%	NCT00876616	362	TAC+MMF+Prednisone	4mg/d	CTX+prednisone	24
Zheng Z, 2022 (37)	China	34.2	87.60%	NCT02457221	314	TAC+prednisone	4mg/d	CTX+prednisone	24
Bao H, 2008 (25)	China	28.9	90%	NCT00298506	40	TAC+ MMF+prednisone	4mg/d	CTX+prednisone	72
Miyasaka N, 2009 (30)	Japan	36.6	82.50%	NCT00429377	63	TAC+prednisone	3 mg/d	prednisone	28
Mok CC, 2014 (31)	China	35.5	92%	NCT00371319	150	TAC+prednisolone	0.06-0.1 mg/kg/d	MMF+prednisolone	24
Yap DY, 2012 (36)	China	38.3	62.50%	——	16	TAC+prednisolone	0.75–1 g b.i.d.	MMF+prednisolone	96
Chen W, 2012 (26)	China	31.9	87.10%	NCT00615173	70	TAC+prednisolone	Trough blood concentration of 4–6 ng/mL	AZA+prednisolone	24
Li X, 2012 (28)	China	29.5	86.70%	——	60	TAC/CTX+prednisone	0.08–0.1 mg/kg/d	MMF+prednisone	24
Chen W, 2010 (35)	China	32.0	85.20%	NCT00615173	81	TAC+prednisone	0.05-0.1mg/kg/d	CTX+prednisone	24
Zavada J, 2010 (27)	The Czech Republic and Slovakia.	29.0	72.50%	NCT00976300	40	CsA+methylprednisolone	4–5 mg/kg/day	CTX+methylprednisolone	72
Moroni G, 2006 (33)	Italy	31.5	89.90%	——	75	CsA+prednisone	3.5 ± 0.5 mg/kg/d (range 2.5 to 4.3)	AZA+prednisone	96
El-Sehemy MS, 2006 (32)	Egypt	22.0	100%	——	22	CsA/AZA+methyl prednisolone	Sarting with 1–2 mg/kg/d	CTX+methyl prednisolone	24
Howard A. Austin, (24)	America	40.0	83.30%	——	42	CsA/CTX+prednisolone	About 5 mg/kg per day	prednisone	48
Z. Haitao, 2006 (38)	China	30.0	100%	——	37	TAC+prednisolone	The initial dose is 0.1 mg/(kg·d)	CTX+prednisolone	24

N, Total number of patients; bid, twice a day; CsA, Cyclosporine A; TAC, Tacrolimus; MMF, mycophenolate mofetil; AZA, azathioprin; CTX, Cyclophosphamide.

### 3.2 Risk of bias

**Figure 2:**
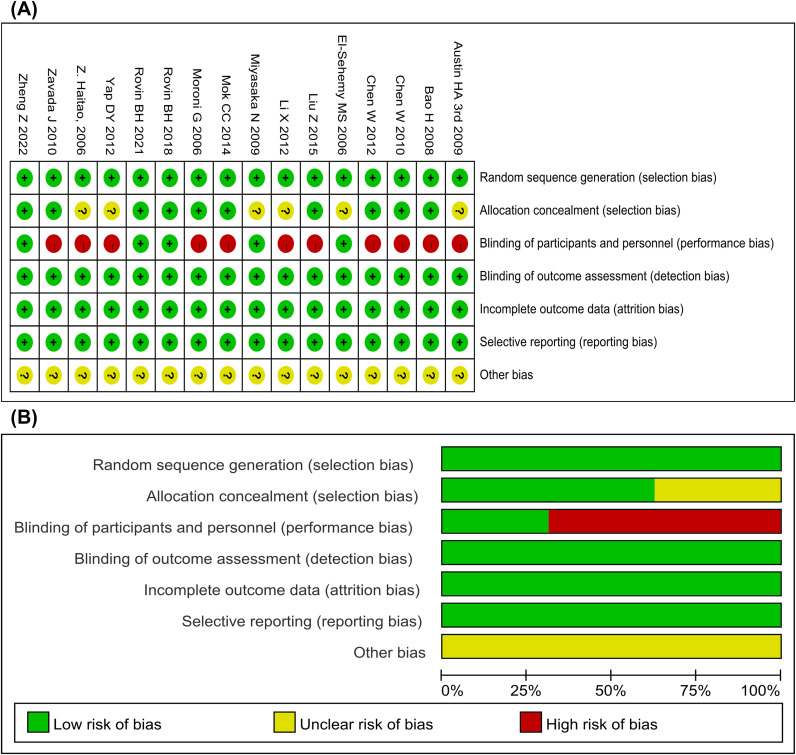
Assessment of risk of bias. **(A)** Risk of bias summary for all included study. **(B)** Risk of bias graph for all included studies.

All RCTs employed randomization techniques and most of the included RCTs provided details regarding the concealment of the allocation sequence, and reporting bias was effectively addressed. As shown in **Figure 2**, 10 studies detailed the sequence generation, marked as “low risk”. Five studies clarified the distribution concealment, indicating blindness for both participants and researchers, and marked it as “low risk”. All other biases in the RCTs were marked as “unclear”. Overall, most of the trials were of low-to-moderate quality.

### 3.3 Heterogeneity assessment

This study utilized the Mantel-Haenszel method to pool effect sizes for different outcomes. Heterogeneity analysis was performed for the outcomes of CNI versus standard therapy using the I² statistic and Chi² test. When P ≥ 0.10 and I^2^ ≤ 50%, we adopted a fixed-effects model; if P < 0.10 or I^2^ > 50%, we switched to a random-effects model.

**Figure 3:**
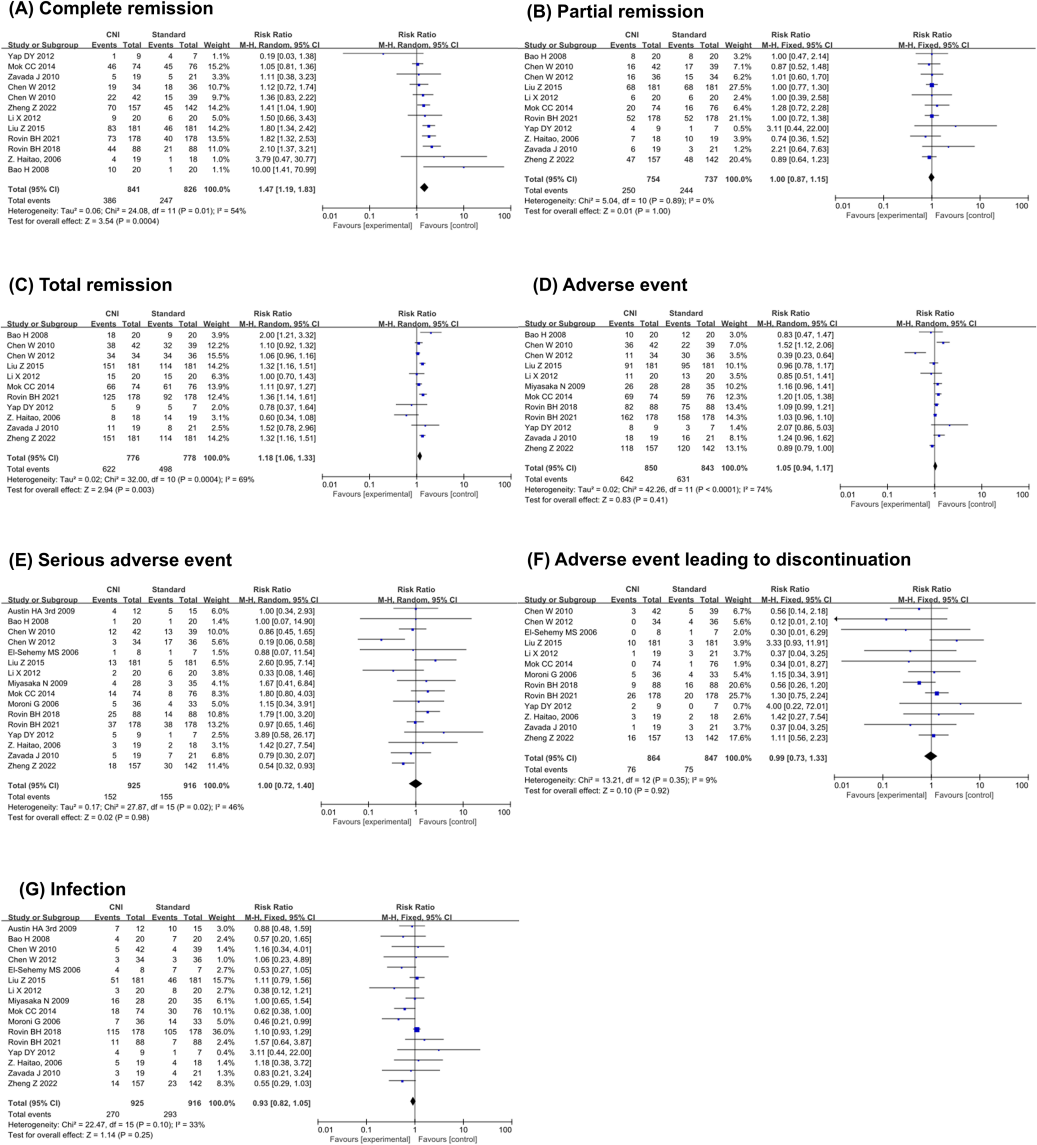
Forest plots of efficacy and safety indicators. **(A)** Complete remission, **(B)** Partial remission, **(C)** Total remission, **(D)** Adverse event, **(E)** Serious adverse event, **(F)** Adverse event leading to discontinuation, **(G)** Infection. CNI: calcineurin inhibitors.

As shown in **Figure 3**, for efficacy-related outcomes, high heterogeneity was observed for CR (I² = 54%; Chi² = 24.08; P = 0.01) and TR (I² = 69%; Chi² = 32.00; P = 0.0004), while PR exhibited low heterogeneity (I² = 0%; Chi² =5.04; P = 0.89). Regarding adverse events, substantial heterogeneity was detected (I² = 74%; Chi² = 42.26; P < 0.0001). In contrast, SAE, infection, and adverse events leading to discontinuation showed low to moderate heterogeneity (SAE: I² =46%, Chi² = 27.87, P = 0.02; AE leading to discontinuation: I² = 9%, Chi² = 13.21, P = 0.35; infection: I² = 33%, Chi² = 22.47, P = 0.10). For the outcomes of CR, TR, AE, and SAE, a random-effects model was employed for the NMA, whereas a fixed-effect model was used for PR, infection, and AE leading to discontinuation.

### 3.4 Inconsistency tests

The global inconsistency test of CR, PR, TR, AE, SAE, AE leading to discontinuation and infection showed that *P* values were 0.9633, 0.7359, 0.7025, 0.3476, 0.1677, 0.3655 and 0.7865 respectively. All *P* values were > 0.05, indicated that the global inconsistency was not significant. The node-splitting analysis, presented in **Supplementary Figure S1**, showed that the results of direct and indirect comparisons were consistent across all cases (all *P*-values > 0.05). The obtained forest plot (**Supplementary Figure S2**) displays the comparisons between direct and indirect evidence for all treatment contrasts within the network. Across all presented contrasts, the confidence intervals for both direct and indirect effects show substantial overlap, and no comparison indicates a statistically significant difference between the two sources of evidence. This finding is in agreement with the results of the global consistency test, collectively demonstrating the reliability of the findings from this NMA.

### 3.5 Efficacy analysis

#### 3.5.1 Complete remission rate

**Figure 4:**
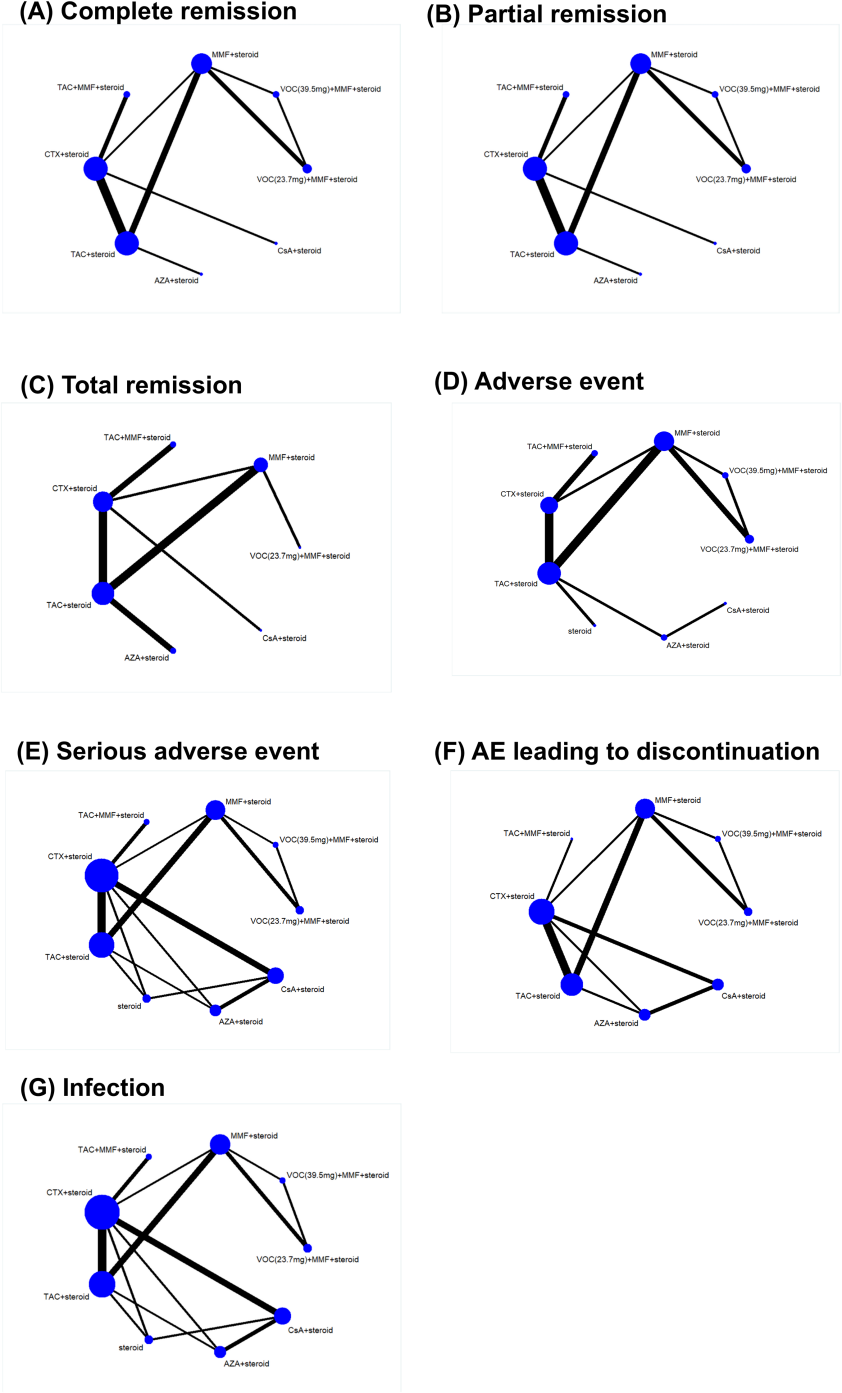
The network evidence map of efficacy and safety indicators. The size of each node is proportional to the sample size of the individual treatment regimen; the widths of the connecting lines are proportional to the number of studies directly comparing the two connected regimens. **(A)** Complete remission, **(B)** Partial remission, **(C)** Total remission, **(D)** Adverse event, **(E)** Serious adverse event, **(F)** AE leading to discontinuation, **(G)** Infection. AE, adverse event; VOC, voclosporin; MMF, mycophenolate; TAC, tacrolimus; CTX, cyclophosphamide; AZA, azathioprine; CsA, cyclosporine A.

Of the included studies, twelve involving 1792 patients described the outcomes of CR and included a total of eight interventions. The network evidence map is presented in **Figures 4A-C**. In the network diagram for CR, the thickness of connecting lines corresponds to the number of studies directly comparing pairs of interventions. Interventions without connecting lines were compared indirectly via NMA. The size of nodes represents the total sample size for each intervention. Results indicate that the comparison between TAC + steroid and CTX + steroid had the highest number of direct studies, while CsA + steroid versus CTX + steroid had the fewest studies.

The probability ranking of treatment protocols based on SUCRA values is shown in **Table 2**. Detailed information can be found in **Supplementary Table S2**. SUCRA values range from 0 to 100, with higher values (closer to 100) indicated greater effectiveness of the intervention. For the efficacy indicator of CR, the rankings of the top three were as follows: VOC(23.7mg) + MMF + steroid (SUCRA = 96.6%) > VOC(39.5mg) + MMF + steroid (SUCRA = 79.3%) > TAC + MMF + steroid (SUCRA = 70.7%). Across all intervention protocols, the CTX + steroid combination demonstrated the most limited therapeutic efficacy (SUCRA = 9.0%). For a comprehensive visualization of treatment hierarchies, the cumulative probability plots for all efficacy outcomes (CR, PR, and TR) are provided in **Supplementary Figure S3**.

**Table 2:** Rank probability of efficacy for each treatment invention.

Intervention measure	CR		PR		TR	
	SUCRA/%	Rank	SUCRA/%	Rank	SUCRA/%	Rank
VOC(23.7mg)+MMF+steroid	96.6	1	22.4	6	84.3	1
VOC(39.5mg)+MMF+steroid	79.3	2	/	/	/	/
MMF+steroid	40.9	5	19.4	7	22.4	6
TAC+MMF+steroid	70.7	3	57.9	4	78.0	2
CTX+steroid	9.0	8	59.9	2	8.4	7
TAC+steroid	42.9	4	41.1	5	48.4	4
AZA+steroid	32.4	6	58.4	3	32.2	5
CsA+steroid	28.1	7	90.9	1	76.2	3

‘/’ means that this treatment plan was not included in this analysis. VOC, voclosporin; MMF, mycophenolate; TAC, tacrolimus; CTX, cyclophosphamide; AZA, azathioprine; CsA, cyclosporine A.

**Figure 5:**
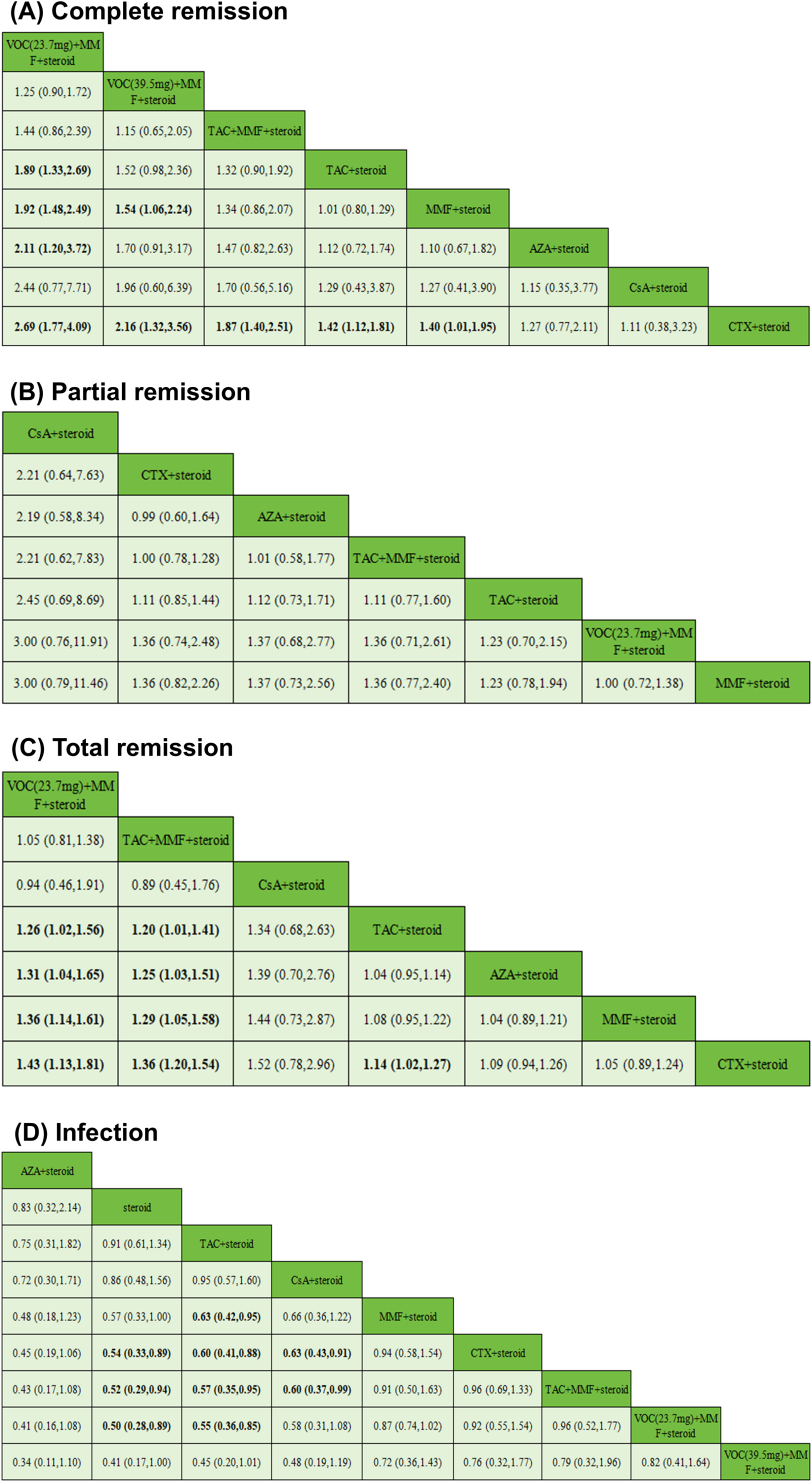
Comparison of the therapeutic effects of the regimens. The RR and 95% CI for comparison of the efficacy of each treatment regimen are provided. An RR >1 indicates that the treatment on the top left is better than the comparative treatment. The bold numbers indicate statistical significance. **(A)** Complete remission, **(B)** Partial remission, **(C)** Total remission, **(D)** Infection. VOC, voclosporin; MMF, mycophenolate; TAC, tacrolimus; CTX, cyclophosphamide; AZA, azathioprine; CsA, cyclosporine A.

VOC(23.7 mg) + MMF + steroid was significantly more effective than TAC+steroid (RR = 1.89, 95%CI = 1.33-2.69), MMF + steroid (RR = 1.92, 95%CI = 1.48-2.49), AZA+steroid (RR = 2.11, 95%CI = 1.20-3.72), and CTX+steroid (RR = 2.69, 95%CI = 1.77-4.09). The above differences were statistically significant (**Figure 5A**).

**Figure 6:**
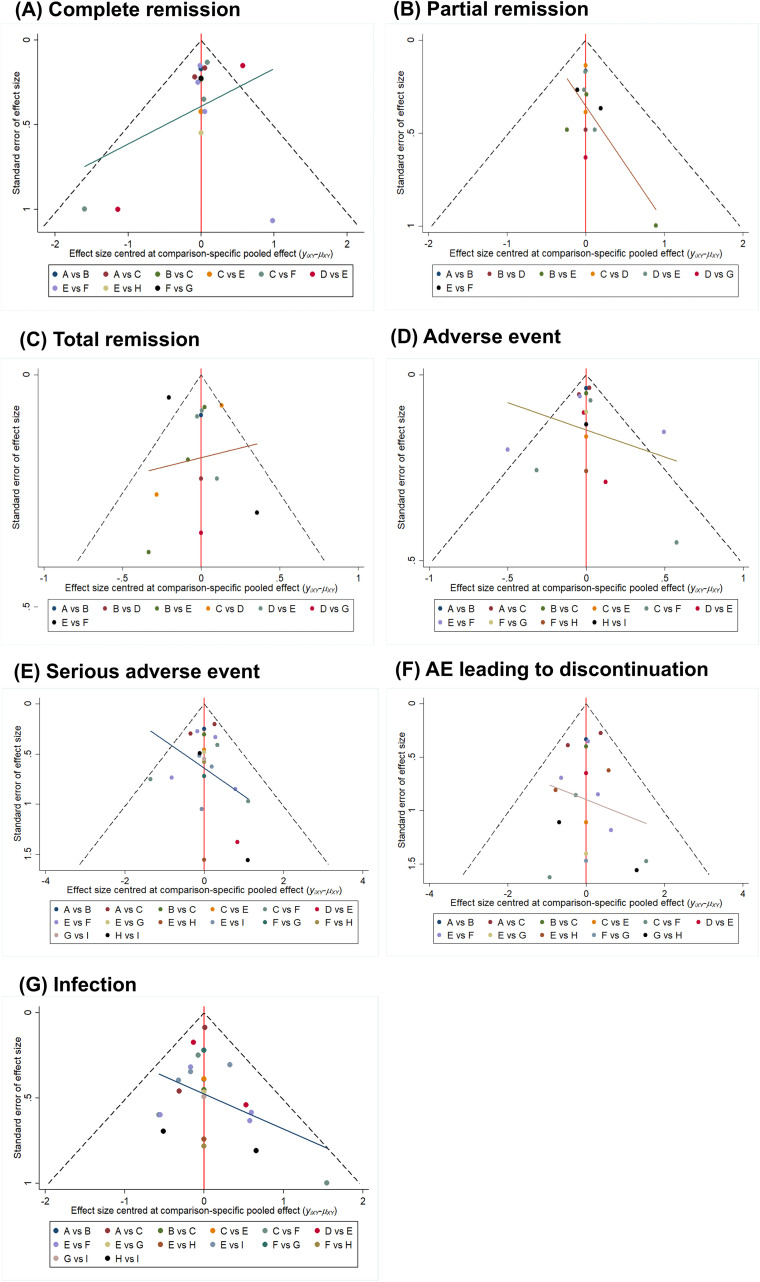
Funnel plot for efficacy and safety outcomes. AE, adverse event; In picture **(A, F)**: A: voclosporin(23.7 mg)+mycophenolate mofetil+steroid; B: voclosporin(39.5 mg)+mycophenolate mofetil+steroid; C: mycophenolate mofetil+steroid; D: tacrolimus+mycophenolate mofetil+steroid; E: cyclophosphamide + steroid; F: tacrolimus+steroid; G: azathioprine+steroid; H: cyclosporine A+ steroid. In picture **(B, C)**: A: voclosporin(23.7 mg)+mycophenolate mofetil+steroid; B: mycophenolate mofetil+steroid; C: tacrolimus+mycophenolate mofetil+steroid; D: cyclophosphamide+steroid; E: tacrolimus+steroid; F: azathioprine+steroid; G: cyclosporine A+ steroid. In picture **(D, E, G)**: A: voclosporin(23.7 mg)+mycophenolate mofetil+steroid; B: voclosporin(39.5 mg)+mycophenolate mofetil+steroid; C: mycophenolate mofetil+steroid; D: tacrolimus+mycophenolate mofetil+steroid; E: cyclophosphamide+steroid; F:tacrolimus+steroid; G:steroid; H: azathioprine+steroid; I: cyclosporine A+steroid.

Funnel plots and Egger’s test were used to assess publication bias for the key outcomes. For CR, neither visual examination nor Egger’s test (P = 0.746) found significant evidence of publication bias (**Figure 6A**).

#### 3.5.2 Partial remission rate

The network evidence map of the PR rate in 11 trials is shown in **Figure 4B**. Notably, the pairwise comparisons did not show statistically significant differences between the treatment regimens (**Figure 5B**). Concurrently, the SUCRA ranking indicated that CsA+steroid had the highest probability of being the most effective treatment (SUCRA = 90.9%).However, the interpretation of this ranking is severely limited by the presence of significant publication bias, as indicated by Egger’s test (P = 0.026, **Figure 6B**). This bias undermines the reliability of the network estimates from which the SUCRA values are derived. Therefore, the ranking should be viewed as unstable and interpreted with extreme caution.

#### 3.5.3 Total remission rate

For the efficacy indicator of TR, the rankings of the top three were as follows: VOC(23.7mg) + MMF+steroid (SUCRA = 84.3%) > TAC + MMF + steroid (SUCRA = 78.0%) > CsA+steroid (SUCRA = 76.2%).

The TR rate of TAC + steroid (RR = 1.26, 95%CI = 1.02-1.56), AZA + steroid (RR = 1.31, 95%CI = 1.04-1.65), MMF + steroid (RR = 1.36, 95%CI = 1.14-1.61), CTX + steroid (RR = 1.43, 95%CI = 1.13-1.81) was found to be lower than that of VOC(23.7 mg) + MMF+steroid. Besides, the TR rate of TAC + steroid (RR = 1.20, 95%CI = 1.01-1.41), AZA + steroid (RR = 1.25, 95%CI = 1.03-1.51), MMF + steroid (RR = 1.29, 95%CI = 1.05-1.58), CTX + steroid (RR = 1.36, 95%CI = 1.20-1.54) was found to be lower than that of TAC + MMF + steroid. The difference was statistically significant. (**Figure 5C**). For TR, neither visual examination nor Egger’s test (P = 0.799) found significant evidence of publication bias (**Figure 6C**).

### 3.6 Safety analysis

Sixteen studies involving 1994 patients assessed safety outcomes across nine interventions. The network evidence map is presented in **Figures 4D-G**. In the NMA, league tables displayed the comparative safety profiles of all investigated drugs and reported RR with corresponding 95% credible intervals.

Based on SUCRA values, MMF + steroid ranked lowest in AE and SAE incidence, while CsA + steroid ranked lowest in discontinuations due to AE (**Table 3**). Detailed information can be found in **Supplementary Table S3**. However, no significant intergroup differences were observed in the incidence of AE, SAE, AE leading to discontinuation among the intervention groups (**Supplementary Figure S5**). Among the three CNIs, the incidence of infection decreased in the following order: VOC(39.5 mg) + MMF + steroid > VOC(23.7 mg) + MMF + steroid > TAC + MMF + steroid > CsA + steroid > TAC + steroid (**Table 3**).

**Table 3:** Rank probability of safety for each treatment invention.

Intervention measure	AE		SAE		AE leading to discontinuation		Infection	
	SUCRA/%	Rank	SUCRA/%	Rank	SUCRA/%	Rank	SUCRA/%	Rank
VOC(23.7mg)+MMF+steroid	73.0	2	66.5	4	42.8	6	20.6	8
VOC(39.5mg)+MMF+steroid	66.8	4	66.3	3	40.2	7	14.5	9
MMF+steroid	82.0	1	85.9	1	48.3	5	39.7	5
TAC+MMF+steroid	56.0	5	9.5	9	14.6	8	27.0	7
CTX+steroid	42.5	7	40.0	6	60.9	3	30.5	6
TAC+steroid	49.4	6	68.8	2	64.2	2	76.0	3
steroid	67.1	3	64.0	5	/	/	83.2	2
AZA+steroid	10.2	8	12.3	8	48.7	4	86.7	1
CsA+steroid	3.1	9	36.8	7	80.4	1	71.7	4

‘/’ means that this treatment plan was not included in this analysis. VOC, voclosporin; MMF, mycophenolate; TAC, tacrolimus; CTX, cyclophosphamide; AZA, azathioprine; CsA, cyclosporine A.

The infection rates of MMF+steroid (RR = 0.63, 95%CI = 0.42-0.95), CTX + steroid(RR = 0.60, 95%CI = 0.41-0.88), TAC + MMF + steroid (RR = 0.57, 95%CI = 0.35-0.95), VOC(23.7mg) + MMF + steroid (RR = 0.55, 95%CI = 0.36-0.85) were found to be higher than that of TAC + steroid (**Figure 5D**). The difference was statistically significant.

Funnel plots for the safety indicators (AE, SAE, AE leading to discontinuation, and infection) showed a generally symmetrical distribution of study points, suggesting no obvious visual evidence of publication bias (**Figure 6**). This subjective impression was quantitatively corroborated by Egger’s regression tests, which yielded non-significant P-values for all safety outcomes: AE (P = 0.147), SAE (P = 0.587), AE leading to discontinuation (P = 0.533), and Infection (P = 0.574). Together, these findings indicate no statistically significant evidence of publication bias for the collected safety data.

### 3.7 Sensitivity analysis

To verify the robustness of the meta-analysis results and to explore potential sources of heterogeneity, a leave-one-out sensitivity analysis was employed. This procedure involved systematically removing each study one at a time and recalculating the pooled effect size.

The analysis was conducted on the following: (1) all primary outcome measures (e.g., total remission rate and complete remission rate); and (2) secondary outcome measures that exhibited substantial heterogeneity (I² ≥ 50%), such as the incidence of adverse events. For outcomes with low heterogeneity (I² < 50%), including partial remission rate and incidence of infection, no sensitivity analysis was performed as the included studies demonstrated good consistency, suggesting a limited potential for any single study to disproportionately influence the pooled results.

The results confirmed the robustness of the findings for most outcomes, as detailed in the supplementary tables (TR: **Supplementary Table S4**; AE: **Supplementary Table S5**). Specifically, the re-estimated effect sizes for TR and AE showed only minor fluctuations, all of which remained within the 95% confidence interval of the overall model. This indicates that no single study exerted a substantial influence on these pooled findings.

In the case of CR, which initially presented with heterogeneity, the sensitivity analysis (**Supplementary Table S6**) nonetheless confirmed the robustness of the primary conclusion. The exclusion of a single study (31), identified as the major source of heterogeneity, led to a substantial reduction in heterogeneity to a non-significant level(I^2^ = 36%, P = 0.11). Furthermore, the intervention then demonstrated a larger and more consistent benefit (RR = 1.57, 95%CI = 1.29-1.92). This finding strengthens the reliable conclusion that the intervention is superior to the control for the CR outcome.

### 3.8 Cluster analysis

**Figure 7:**
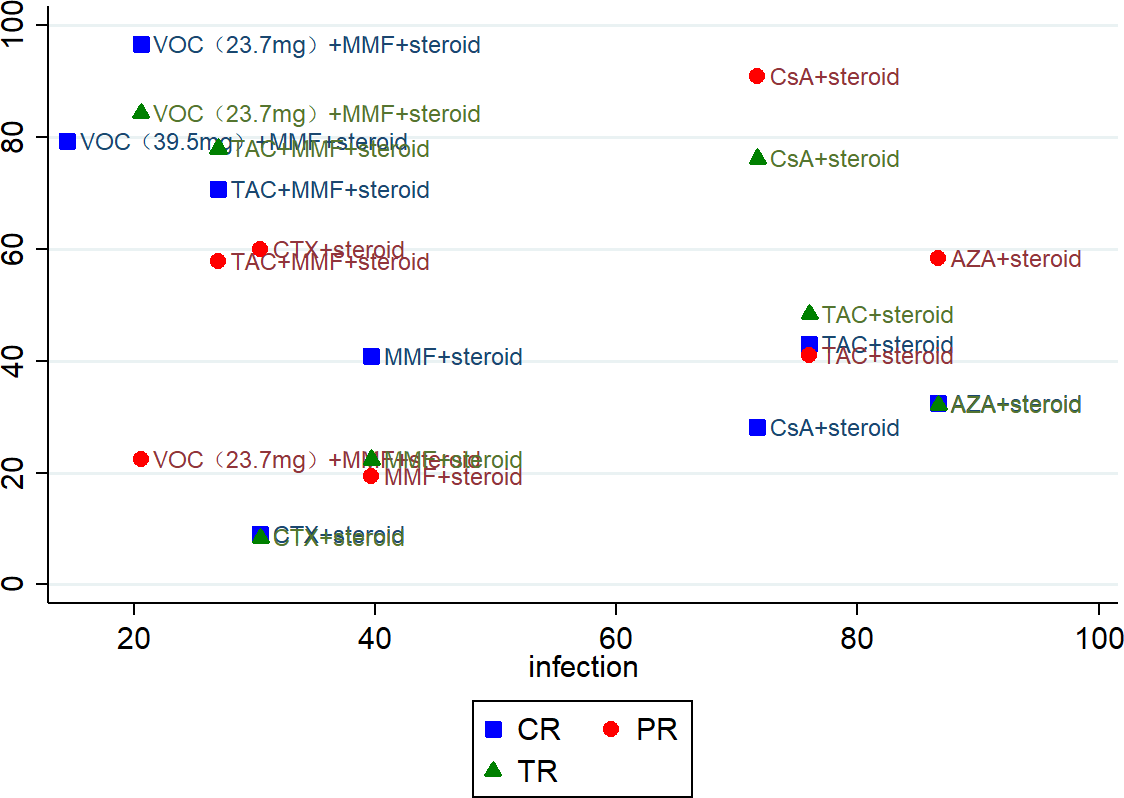
Clustered ranking plot of efficacy and safety outcomes. CR, complete remission; PR, partial remission; TR, total remission. Note: The safety outcome presented in this figure is infection, which was the only safety outcome that showed statistically significant differences in the network meta-analysis.

Treatments were ranked using SUCRA (**Figure 7**). The vertical axis (Y-axis) represents composite efficacy, comprising the endpoints: CR, PR, and TR. The horizontal axis (X-axis) indicates the safety score, where higher values denote a more favorable safety profile. VOC demonstrated the highest efficacy in achieving CR and TR (with highest SUCRA) but conversely showed the poorest safety profile regarding infection risk (with lowest SUCRA). Although CsA demonstrated poorer efficacy in CR/TR, it showed a lower risk of infections compared to other regimens.

## 4 Discussion

Sixteen RCTs with a total of 1994 patients met the inclusion criteria. This NMA compared the relative efficacy and safety of calcineurin inhibitors and therapy as CTX/MMF/AZA for LN. NMA combines both direct and indirect evidence, offering a more comprehensive exploration of the relative effects of different treatment options compared to traditional meta-analysis (39).

The 2024 KDIGO Lupus Nephritis Guidelines and the EULAR 2023 SLE Guidelines both highlighted the importance of CNI in treating LN. Current clinical guidelines recommend the integration of VOC, a next-generation CNI, into the therapeutic strategy for active LN, primarily due to its potent inhibition of calcineurin phosphatase. This mechanism selectively blocks the dephosphorylation and nuclear translocation of NFAT, thereby suppressing the transcription of proinflammatory cytokines such as IL-2, interleukin-4 (IL-4), and TNF-α. Moreover, for active type III/IV ± V LN, especially in patients without severe impairment of renal function (eGFR > 45 mL/min/1.73 m²), multitargeted therapy (glucocorticoids + MMF + TAC) is recommended as the first-line induction therapy.

Building upon this broad consensus and specifically aligning with the latest authoritative guidance, our findings should be interpreted in the context of the recently updated 2024 EULAR/ACR recommendations for the management of lupus nephritis (11, 40). These guidelines significantly elevate the position of CNIs, recommending VOC or TAC in combination with MMF and glucocorticoids as one of the first-line options for the initial treatment of class III/IV (± V) LN. Our NMA provides robust quantitative evidence that strongly supports this paradigm shift. We have demonstrated that treatment regimens based on CNIs, particularly VOC + MMF + glucocorticoids and TAC + MMF + glucocorticoids, rank highly in achieving CR and TR, which is consistent with the significant advantages of CNIs in rapidly reducing protein leakage. Furthermore, our comprehensive safety profile assessment, which showed a manageable safety profile for CNIs without significant increase in major adverse events compared to CTX, addresses potential concerns and reinforces the guideline’s recommendation. By systematically comparing and ranking multiple CNI-containing strategies within a network, our study offers a nuanced evidence base that can aid clinicians in selecting among the guideline-endorsed CNI options tailored to individual patient profiles.

Prior research has yet to fully explore the clinical effectiveness of VOC. To bridge this gap, we designed a comparative analysis with two dosage cohorts (23.7 mg and 39.5 mg) to evaluate its efficacy and safety across varying doses. Our research findings indicated that the combination of VOC and MMF and steroid achieved the highest CR and TR, followed by TAC multitarget therapy. This might be due to the pharmacological properties of VOC: more stable plasma drug concentration, and stronger calcineurin inhibition efficacy (41). Even though the treatment regimen of TAC + MMF + steroid ranks after VOC, it is still an efficient regimen. These findings align with a previous meta-analysis, comparing immunosuppressants, which showed that the combination of VOC with MMF and steroid achieves the highest CR rate (21). CsA ranked last in terms of CR. It is crucial to interpret this finding with caution, as only a single study on CsA was included in the CNI effectiveness analysis, limiting the robustness of this ranking. Notably, although CsA-based regimens were the least effective in achieving CR, they remained widely used in clinical practice due to their long-term safety (greater than 20 years) and cost-effectiveness in resource-limited settings (42). Besides, as all studies included in the multitarget therapy analysis were performed in Chinese patients, additional research is essential to assess whether the favorable outcomes of multitarget therapy can be reproduced in LN patients from non-Asian populations.

This study evaluated the safety profiles of immunosuppressive regimens for SLE, focusing on four key outcomes: AE, SAE, AE leading to discontinuation, and infection events. Studies have shown that infection is the leading cause of death in SLE patients (43). Among the evaluated immunosuppressive regimens, the VOC-MMF-steroid combination demonstrated the highest incidence of infections, and this increased risk was statistically significant. Based on the cumulative ranking probability derived from the SUCRA analysis, TAC-based multi-target therapy demonstrated the least favorable safety profile for SAE, ranking lowest in SAE occurrence with a SUCRA value of 9.5%. However, our research showed that there were no statistically significant differences among the treatment regimens in terms of AE, SAE, and AE leading to discontinuation. This might be due to the limited number of included trials. Although SUCRA values provided a quantitative approach to ranking, we acknowledged that all ranking methods carry some degree of uncertainty. This uncertainty may arise from factors including data quality, study design, heterogeneity among studies, and potential publication bias.

This study assessed the efficacy-safety profiles of different immunosuppressant and corticosteroid combination regimens via visualization using cluster ranking plots. Based on the available indirect evidence, the VOC + MMF + steroid regimen appeared to rank highest in terms of efficacy for inducing remission (CR/TR). However, this regimen was also associated with the highest risk of infection. TAC + MMF + steroid (multitarget therapy) is a highly effective, guideline-recommended first-line regimen. Its efficacy approaches that of the VOC regimen, with a moderate infection risk, and has substantial supporting evidence in Asian populations. CsA + steroid ranks lower in terms of efficacy, yet it offers a relatively lower infection risk. It retains significant value due to its cost-effectiveness, accessibility, and utility in specific clinical scenarios. For patients at high risk of infection (e.g., those with leukopenia, comorbid diabetes, or chronic infections), the selection of high-efficacy regimens requires more careful consideration. Conversely, for severely ill patients pursuing rapid and deep remission, accepting this risk may be clinically justified. These findings suggest that clinicians should integrate drug-specific efficacy profiles with patient-specific clinical characteristics (e.g., comorbidities, risk tolerance) to optimize therapeutic decision-making.

This NMA demonstrated methodological rigor through four key advancements: (1) all included studies were RCTs, which may eliminate selection and confounding biases related to observational studies; (2) the integration of updated statistical metrics, including SAE and discontinuation rates, refined safety evaluations; (3) the inclusion of a broader range of research compared to prior studies, thereby enhanced the robustness and generalizability of the findings; and (4) risk (i.e., infection) and benefit (i.e., efficacy) were also considered simultaneously using clustered ranking plot.

However, this study has several limitations that warrant consideration. First, the generalizability of our findings may be constrained by the overrepresentation of Asian populations in the included trials, as ethnic differences in allele frequencies of pharmacogenomic determinants—particularly CYP3A5 polymorphisms—can significantly impact drug exposure (e.g., bioavailability and clearance) and thus efficacy and safety (44, 45). Specifically, TAC pharmacokinetics are highly CYP3A5 genotype-dependent, requiring higher doses in expressers (a phenotype common in individuals of African descent), which limits the cross-ethnic generalizability of its dosing strategies and outcomes. In contrast, VOC exhibits a genotype-independent metabolic profile, potentially offering more predictable pharmacokinetic and pharmacodynamic responses and stronger external validity to genetically diverse populations. Therefore, the treatment effects and optimal dosing strategies identified here may not be directly applicable to populations with differing genetic backgrounds.

Second, several methodological limitations should be acknowledged. While our search strategy encompassed four major databases and one clinical trial registry (ClinicalTrials.gov), it did not extend to other major international registries such as the WHO International Clinical Trials Registry Platform (ICTRP) or the EU Clinical Trials Register (EUCTR), nor to gray literature or conference proceedings. This may have resulted in the omission of some relevant unpublished or ongoing studies, potentially introducing publication bias. Moreover, the number of randomized controlled trials available was limited, and some had relatively small sample sizes, which consequently precluded planned subgroup analyses to further investigate the sources of heterogeneity. In addition, as many comparisons in the NMA relied on indirect evidence, potential biases cannot be ruled out, and results should be interpreted with caution. Furthermore, not all studies reported every outcome of interest, which may have led to incomplete analysis for certain endpoints.

Third, the structure of the NMA itself was a key source of heterogeneity. We treated “CNI” as a single node despite pharmacokinetic differences between VOC, TAC, and CsA, introducing within-class heterogeneity. To resolve this in future research, we recommend that network meta-analyses treat VOC, TAC, and CsA as distinct nodes when sufficient head-to-head comparative data become available, in order to better quantify their comparative effectiveness and safety, thereby reducing within-class heterogeneity and enhancing the precision of treatment rankings. Additionally, the control group comprised diverse active comparators (e.g., MMF, CTX) with distinct efficacy and safety profiles, which acted as a primary amplifier of statistical heterogeneity. The observed heterogeneity for the complete remission (CR) outcome appeared to be primarily driven by the study by Mok et al. (2014) (31), which was an open-label, phase 4 pragmatic trial. The distinctive features of this trial—namely, its enrollment of a patient population more reflective of real-world practice and the protocol-allowed flexible dose adjustments—may explain its divergent results. This highlights that the efficacy observed in highly controlled efficacy trials (phase 3) might differ from the effectiveness achieved in routine clinical settings, a crucial consideration for the external validity of our findings. Consequently, findings for outcomes with high heterogeneity warrant cautious interpretation.

Future studies should include more head-to-head trials and place greater emphasis on ethnic and pharmacogenomic factors to validate and extend the present findings.

## Data Availability

The original contributions presented in the study are included in the article/**Supplementary Material**. Further inquiries can be directed to the corresponding authors.
