# Supplementary material for: Efficacy and safety of calcineurin inhibitor therapy in lupus nephritis: a systematic review and network meta-analysis
Source: Front Immunol. 2026 Jan 2;16:1670134. doi: 10.3389/fimmu.2025.1670134 (PMC12808350; doi:10.3389/fimmu.2025.1670134)
Supplement: Supplementary file 1 [file Table1.docx]

**Supplementary Materials**

**Efficacy and safety of calcineurin inhibitor therapy in lupus nephritis: a systematic review and** **network meta-analysis**

Contest

[Supplementary Materials 1](#_Toc18748)

[Supplementary Figure S1. Node-split analysis for multiple clinical outcomes. 2](#_Toc32234)

Supplementary Figure S2. Forest plot for the consistency assessment between direct and indirect evidence.............................................................................................................................3

[Supplementary Figure S3. SUCRA ranking of efficacy indicators. 4](#_Toc5427)

[Supplementary Figure S4. SUCRA ranking of safety indicators. 5](#_Toc9868)

[Supplementary Figure S5. League plots of safety indicators. 6](#_Toc18263)

[Supplementary Table S1. Participant baseline characteristics of included RCTs 7](#_Toc5928)

[Supplementary Table S2. SUCRA values of efficacy outcomes for different interventions 10](#_Toc16139)

[Supplementary Table S3. SUCRA values of safety outcomes for different interventions 11](#_Toc14218)

[Supplementary Table S4 Sensitivity analysis of total remission 12](#_Toc6916)

[Supplementary Table S5 Sensitivity analysis of adverse event 13](#_Toc29026)

[Supplementary Table S6 Sensitivity analysis of complete remission 14](#_Toc29904)

[Supplementary Table S7 PRISMA checklist 15](#_Toc31777)

**
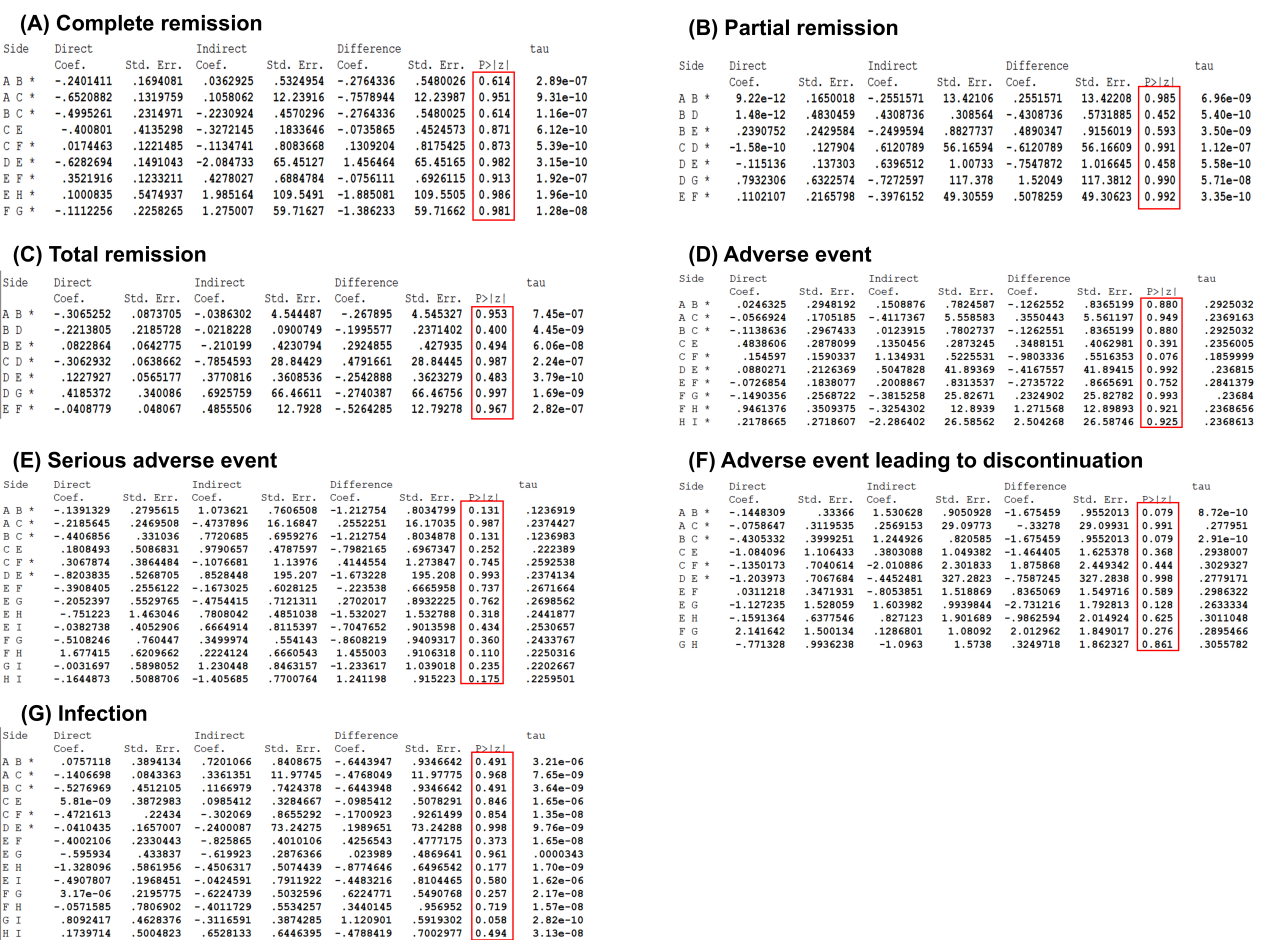
**

# **Supplementary Figure S1. Node-split analysis for** **multiple clinical outcomes.**

**
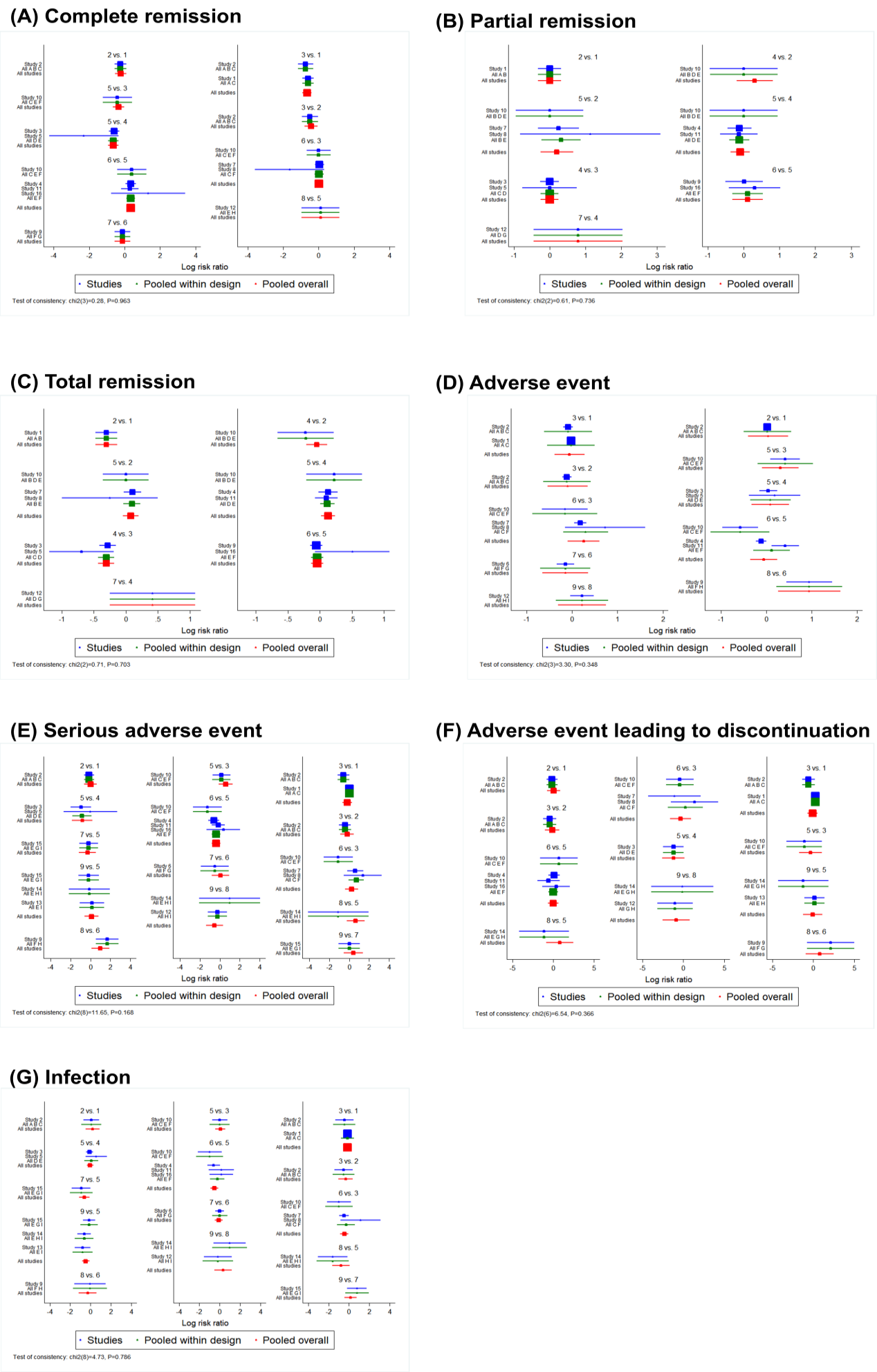
Supplementary Figure S2. Forest plot for the consistency assessment between direct and indirect evidence.**

**
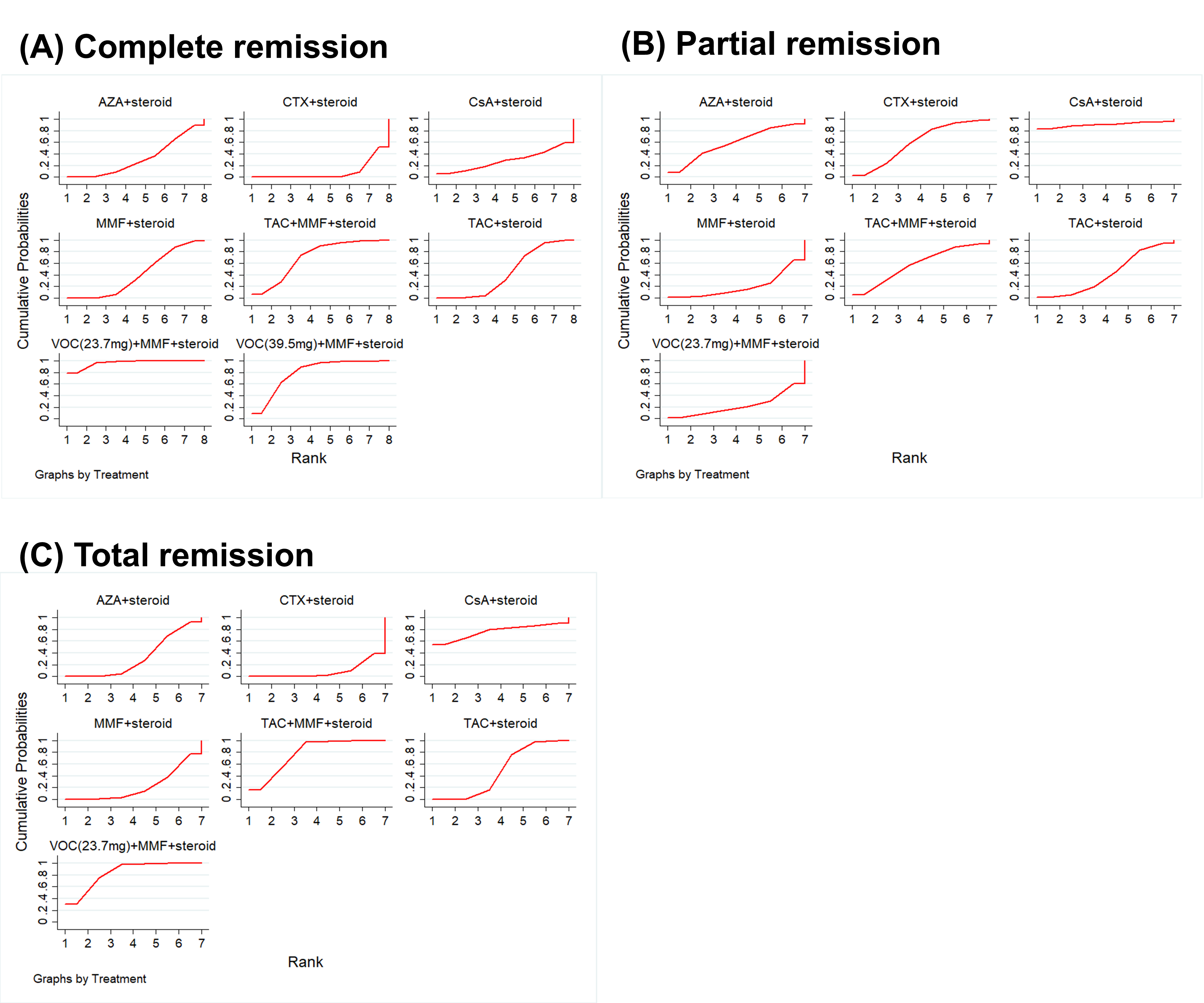
**

# **Supplementary Figure S3. SUCRA ranking of efficacy indicators.**

**
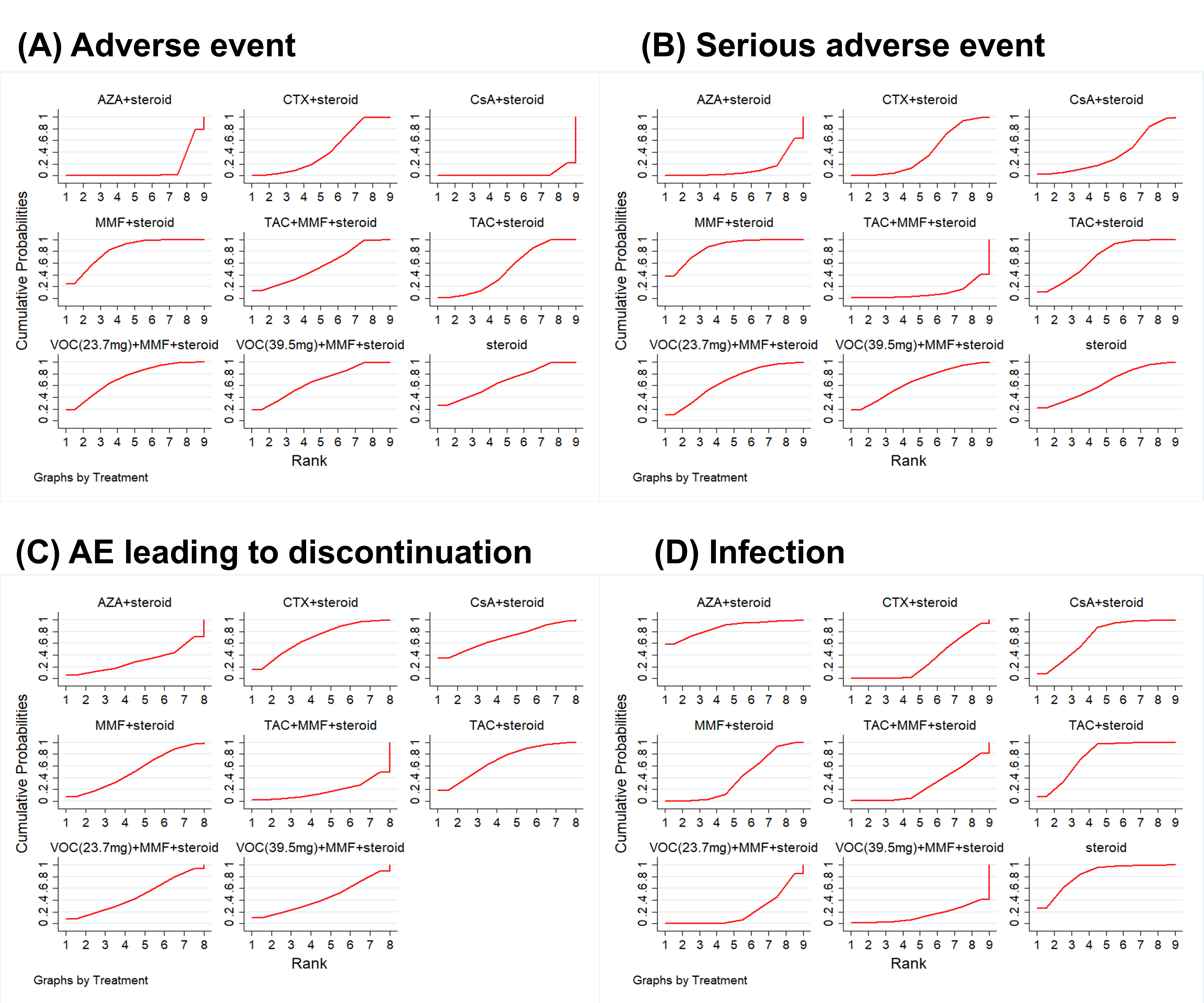
**

# **Supplementary Figure S4. SUCRA ranking of safety indicators.**

AE: Adverse events.


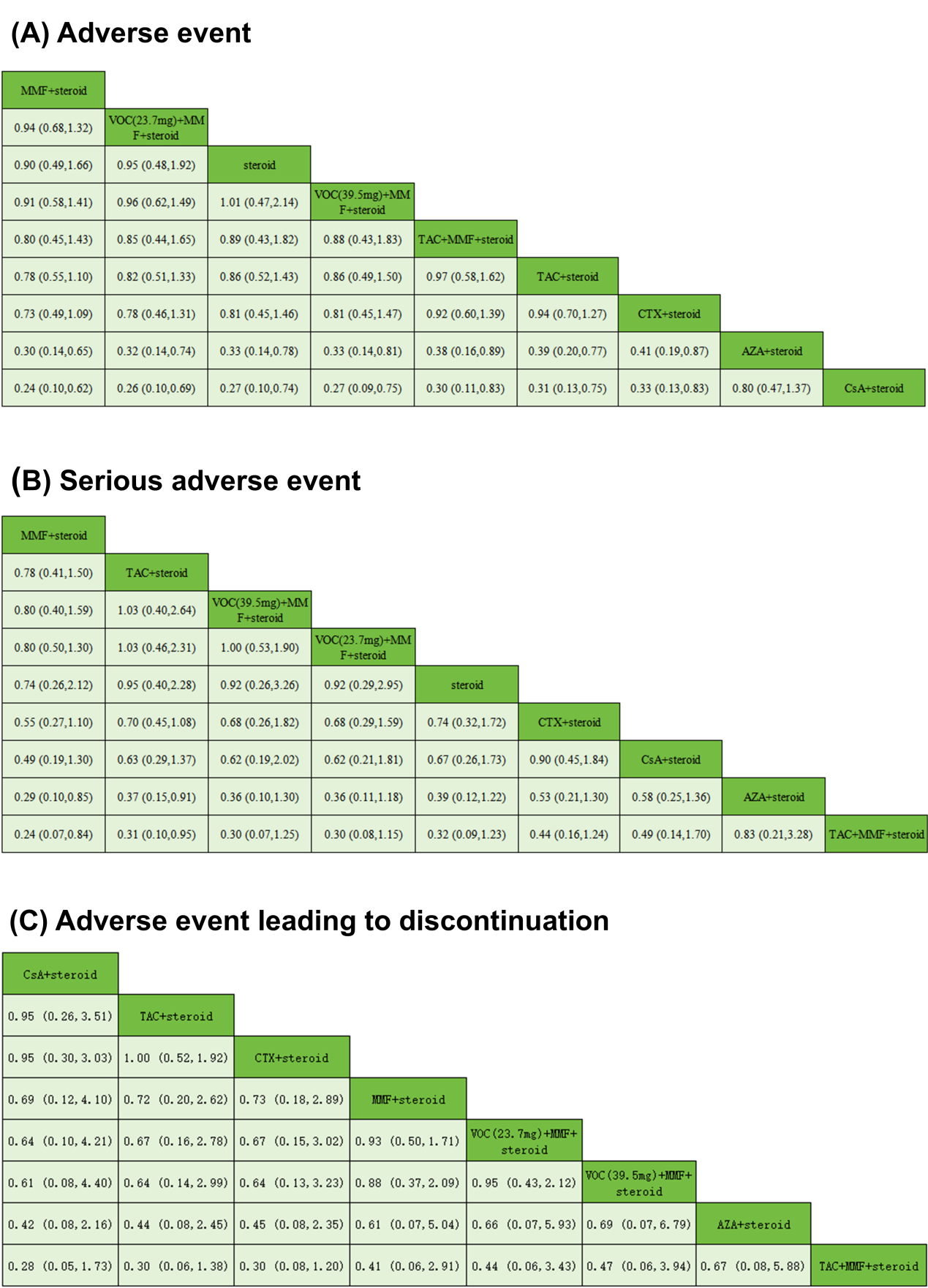


# **Supplementary Figure S5. League plots of safety indicators.**

# **Supplementary Table S1. Participant baseline characteristics of included RCTs**

| **Author, year** | **Group** | **Age，mean**  **(years)** | **disease duration (years)** | **Baseline proteinuria** | **LN class** | **eGFR (mL/min per 1·73 m²)** | **Steroid regimen** | **Ethnicity** |
| --- | --- | --- | --- | --- | --- | --- | --- | --- |
| Rovin BH，2021 | VOC(23.7 mg)  Placebo | 31  32 | 4.6  4.7 | 4.14 mg/mg  3.87 mg/mg | III, IV, V, | 92.1  90.4 | Rapid low-dose taper (to 2.5 mg/d by Wk 16) | **Multiracial** |
| Rovin BH，2019 | VOC(23.7 mg)  VOC(39.5 mg)  Placebo | 31.4  30.6  33.1 | 4.2  3.2  3.5 | 5.16 mg/mg  4.48 mg/mg  4.43 mg/mg | III, IV, V, | 95.3  104.0  100.2 | Rapid low-dose taper (to 2.5 mg/d by Wk 16) | **Multiracial** |
| Liu Z，2015 | TAC  IVCY | 30.3  33.6 | 0.17  0.25 | 3.44 g/24h  3.68 g/24h | **III, IV, V, III+V, IV+V** | 79.2% ≥60 | **IV MP pulse (0.5g/d×3d), then oral Prednisone (0.6 mg/kg/d), tapered to 10 mg/d by Wk24** | **Chinese** |
| Zheng Z，2022 | TAC  IVCY | 34.3  34.1 | 1.4  1.5 | **5.8 g/24h**  **5.3 g/24h** | III、IV、V、III+V、IV+V | 99.4  103.4 | **IV MP (0.5 g/d × 3d) → oral prednisone 0.8 mg/kg/d (max 45 mg/d) × 4 wk, tapered to 10 mg/d by Wk 24** | **Chinese** |
| Bao H，2008 | TAC  IVCY | 27.2  30.6 | **2.5**  **2.2** | 4.41 **g/24h**  4.10 **g/24h** | **V+IV** | 98.6  96.6 | IV MP (0.5 g/d × 3d) → oral prednisone 0.6-0.8 mg/kg/d starting dose, tapered to 10 mg/d maintenance | **Chinese** |
| Miyasaka N，2009 | TAC  Placebo | 37.5  35.5 | 7.8  7.9 | 1.64 **g/24h**  1.47 **g/24h** | Active lupus nephritis | 77.0  76.5 | The glucocorticoid dose could not be increased, but tapering was allowed | Japanese |
| Mok CC，2014 | TAC  MMF | 36.2  36.1 | 4.4  4.0 | 3.32 **g/24h**  3.83 **g/24h** | III, IV, V, | 80.5  77.4 | Oral prednisolone 0.6 mg/kg/day for 6 weeks, then tapered by 5 mg/week to <10 mg/day for long-term maintenance | Chinese |
| Yap DY，2012 | TAC  MMF | 40.0  36.0 | 0.6  1.1 | 4.0 **g/24h**  4.7 **g/24h** | **V** | Not reported | Prednisolone started at 0.8 mg/kg/day (max 50 mg/day), tapered by 5 mg every two weeks to 10 mg/day by ~4 months, then further adjusted for maintenance | Chinese |
| Chen W，2012 | TAC  AZA | 30.7  33.1 | 0.17  0.17 | 0.5 **g/24h**  0.38 **g/24h** | III, IV, V, | 91.1  102.1 | Prednisone 10 mg/day | Chinese |
| Li X，2012 | TAC  IVCY  MMF | 29.0  33.0  26.5 | 0.25  0.17  0.25 | 3.36 **g/24h**  3.70 **g/24h**  3.66 **g/24h** | III、IV、V、V+III、V+IV | 82.2  80.6  96.3 | Initiated at 0.8–1.0 mg/kg/day (max 60 mg/day), tapered to 10 mg/day by ~4 months and maintained | Chinese |
| Chen W，2010 | TAC  IVCY | 32.0  31.9 | 3.8  3.3 | 0.47 **g/24h**  0.34 **g/24h** | III、IV、V、V+III、V+IV | 1.84  1.85 | Initiated at 1.0 mg/kg/day (max 60 mg/day), tapered to 10 mg/day and maintained | Chinese |
| Zavada J，2010 | CsA  IVCY | 28  30 | Not reported | 2.5 **g/24h**  3.8 **g/24h** | III、IV | 91.2  86.9 | Initiated at 0.8 mg/kg/day, tapered to 4 mg/day over 16 months | Caucasian |
| Moroni G，2006 | CsA  AZA | 31.7  31.2 | 5.4  2.3 | 2.8 **g/24h**  2.2 **g/24h** | IV、V | 92.5  104.1 | 3-day IV methylprednisolone pulses, then oral prednisone tapered from 1 to 0.5 mg/kg/day over 2 months | Caucasian |
| El-Sehemy MS ，2006 | CsA  AZA  IVCY | 22.0  21.4  25.6 | 10.3  17.6  0.71 | 6.9 **g/24h**  3.7 **g/24h**  3.0 **g/24h** | IV, V | 23.7  53.6  31.4 | IV methylprednisolone pulse (500–1000 mg/day for 3–5 days), followed by oral prednisolone (0.5 mg/kg/day) tapered to 5–10 mg/day | Egyptian |
| Howard A. Austin,2009 | CsA  IVCY  Prednisone | 41.0  34.0  40.0 | 0.6  0.5  1 | 5.0 **g/24h**  5.8 **g/24h**  5.7 **g/24h** | V | 80.0  89.0  80.0 | All patients received alternate-day oral prednisone: 40 mg/m² (≈1 mg/kg) for 8 weeks, tapered over 1 year to 10 mg/m² | **Multiracial** |
| Z. Haitao, 2006 | TAC  IVCY | 25.7  30.3 | 2.5  1.7 | 5.7 **g/24h**  4.6 **g/24h** | V+IV | Not reported | All patients received oral prednisone 0.6 mg/(kg·d) (max 40 mg/d) with taper to 10 mg/d; 17/37 also received IV methylprednisolone 0.5 g/day for 3 days | Chinese |

IV: IntraVenous; IVCY: IntraVenous CYclophosphamide; MP:MethylPrednisolone; CsA: Cyclosporine A; TAC: Tacrolimus; MMF: mycophenolate mofetil; AZA: azathioprin; CTX: Cyclophosphamide.

# Supplementary Table S2. SUCRA values of efficacy outcomes for different interventions

|  | | CR | | | | PR | | TR | | | |
| --- | --- | --- | --- | --- | --- | --- | --- | --- | --- | --- | --- |
| Treatments | SUCRA | | PrBest | Mean rank | SUCRA | | PrBest | Mean rank | SUCRA | PrBest | Mean rank |
| VOC（23.7mg）+MMF+steroid | 96.6 | | 78.8 | 1.2 | 22.4 | | 1.3 | 5.7 | 84.3 | 32.1 | 1.9 |
| VOC（39.5mg）+MMF+steroid | 79.3 | | 8.0 | 2.4 | / | | / | / | / | / | / |
| MMF+steroid | 40.9 | | 0.0 | 5.1 | 19.4 | | 0.3 | 5.8 | 22.4 | 0.0 | 5.7 |
| TAC+MMF+steroid | 70.7 | | 7.3 | 3.0 | 57.9 | | 4.5 | 3.5 | 78.0 | 14.5 | 2.3 |
| CTX+steroid | 9.0 | | 0.0 | 7.4 | 59.9 | | 2.2 | 3.4 | 8.4 | 0.0 | 6.5 |
| TAC+steroid | 42.9 | | 0.0 | 5.0 | 41.1 | | 0.7 | 4.5 | 48.4 | 0.0 | 4.1 |
| AZA+steroid | 32.4 | | 0.3 | 5.7 | 58.4 | | 7.7 | 3.5 | 32.2 | 0.0 | 5.1 |
| CsA+steroid | 28.1 | | 5.7 | 6.0 | 90.9 | | 83.3 | 1.5 | 76.2 | 53.4 | 2.4 |

‘/’ means that this treatment plan was not included in this analysis.CR, complete remission; PR, partial remission; TR, total remission; VOC, voclosporin; MMF, mycophenolate; TAC, tacrolimus; CTX, cyclophosphamide; AZA, azathioprine; CsA, cyclosporine A.

# **Supplementary Table S3. SUCRA values of safety outcomes for different interventions**

|  | AE | | | SAE | | | AE leading to discontinuation | | | | Infection | | |
| --- | --- | --- | --- | --- | --- | --- | --- | --- | --- | --- | --- | --- | --- |
| Treatments | SUCRA | PrBest | Mean rank | SUCRA | PrBest | Mean rank | | SUCRA | PrBest | Mean rank | SUCRA | PrBest | Mean rank |
| VOC（23.7mg）+MMF+steroid | 73.0 | 18.6 | 3.2 | 66.5 | 9.4 | 3.7 | | 42.8 | 5.9 | 5.0 | 20.6 | 0.0 | 7.4 |
| VOC（39.5mg）+MMF+steroid | 66.8 | 18.5 | 3.7 | 66.3 | 18.0 | 3.7 | | 40.2 | 8.2 | 5.2 | 14.5 | 0.6 | 7.8 |
| MMF+steroid | 82.0 | 24.0 | 2.4 | 85.9 | 37.8 | 2.1 | | 48.3 | 5.8 | 4.6 | 39.7 | 0.0 | 5.8 |
| TAC+MMF+steroid | 56.0 | 12.0 | 4.5 | 9.5 | 0.3 | 8.2 | | 14.6 | 1.3 | 7.0 | 27.0 | 0.1 | 6.8 |
| CTX+steroid | 42.5 | 0.4 | 5.6 | 40.0 | 0.2 | 5.8 | | 60.9 | 5.6 | 3.7 | 30.5 | 0.0 | 6.6 |
| TAC+steroid | 49.4 | 0.6 | 5.0 | 68.8 | 10.0 | 3.5 | | 64.2 | 11.1 | 3.5 | 76.0 | 6.9 | 2.9 |
| steroid | 67.1 | 25.8 | 3.6 | 64.0 | 21.5 | 3.9 | | / | / | / | 83.2 | 26.0 | 2.3 |
| AZA+steroid | 10.2 | 0.0 | 8.2 | 12.3 | 0.2 | 8.0 | | 48.7 | 13.9 | 4.6 | 86.7 | 58.9 | 2.1 |
| CsA+steroid | 3.1 | 0.0 | 8.8 | 36.8 | 2.5 | 6.1 | | 80.4 | 48.1 | 2.4 | 71.7 | 7.5 | 3.3 |

‘/’ means that this treatment plan was not included in this analysis. AE, adverse event; SAE, serious adverse event; VOC, voclosporin; MMF, mycophenolate; TAC, tacrolimus; CTX, cyclophosphamide; AZA, azathioprine; CsA, cyclosporine A

# **Supplementary Table S4. Sensitivity analysis of total remission**

| Excluded Study | Pooled Effect Size (95% CI) | I² Statistic | Heterogeneity P-value | Tau² | Conclusion Changed |
| --- | --- | --- | --- | --- | --- |
| Overall analysi | 1.18 [1.06, 1.33] | 69% | 0.0004 | 0.02 | - |
| Bao H 2008 | 1.16 [1.04, 1.30] | 66% | 0.001 | 0.02 | No |
| Chen W 2010 | 1.20 [1.05, 1.36] | 71% | 0.0003 | 0.02 | No |
| Chen W 2012 | 1.21 [1.09, 1.36] | 56% | 0.02 | 0.01 | No |
| Liu Z 2015 | 1.16 [1.03, 1.32] | 68% | 0.0009 | 0.02 | No |
| Li X 2012 | 1.20 [1.06, 1.35] | 71% | 0.0002 | 0.02 | No |
| Mok CC 2014 | 1.19 [1.05, 1.36] | 71% | 0.0003 | 0.02 | No |
| Rovin BH 2021 | 1.16 [1.03, 1.31] | 68% | 0.001 | 0.02 | No |
| Yap DY 2012 | 1.19 [1.07, 1.34] | 71% | 0.0003 | 0.03 | No |
| Z. Haitao, 2006 | 1.21 [1.08, 1.35] | 68% | 0.0008 | 0.02 | No |
| Zavada J 2010 | 1.18 [1.05, 1.32] | 71% | 0.0003 | 0.02 | No |
| Zheng Z 2022 | 1.16 [1.03, 1.32] | 68% | 0.0009 | 0.02 | No |

# **Supplementary Table S5. Sensitivity analysis of adverse event**

| Excluded Study | Pooled Effect Size (95% CI) | I² Statistic | Heterogeneity P-value | Tau² | Conclusion Changed |
| --- | --- | --- | --- | --- | --- |
| Overall analysi | 1.30 [0.72, 2.32] | 76% | 0.00001 | 0.7 | - |
| Bao H 2008 | 1.38 [0.74, 2.58] | 78% | 0.00001 | 0.75 | No |
| Chen W 2010 | 1.13 [0.63, 2.02] | 74% | 0.0001 | 0.61 | No |
| Chen W 2012 | 1.57 [0.94, 2.60] | 65% | 0.001 | 0.4 | No |
| Liu Z 2015 | 1.40 [0.68, 2.89] | 68%% | 0.00001 | 1.06 | No |
| Li X 2012 | 1.38 [0.74, 2.58] | 78% | 0.00001 | 0.75 | No |
| Miyasaka N 2009 | 1.22 [0.67, 2.23] | 78% | 0.00001 | 0.7 | No |
| Mok CC 2014 | 1.15 [0.64, 2.08] | 75% | 0.0001 | 0.64 | No |
| Rovin BH 2018 | 1.22 [0.66, 2.28] | 77% | 0.00001 | 0.72 | No |
| Rovin BH 2021 | 1.32 [0.68, 2.57] | 78% | 0.00001 | 0.85 | No |
| Yap DY 2012 | 1.19 [0.66, 2.14] | 77% | 0.00001 | 0.66 | No |
| Zavada J 2010 | 1.21 [0.67, 2.19] | 77% | 0.00001 | 0.69 | No |
| Zheng Z 2022 | 1.46 [0.76, 2.79] | 76% | 0.0001 | 0.79 | No |

| **Supplementary Table S6. Sensitivity analysis of complete remission** | | | | | |
| --- | --- | --- | --- | --- | --- |
| Excluded Study | Pooled Effect Size (95% CI) | I² Statistic | Heterogeneity P-value | Tau² | Conclusion Changed |
| Overall analysi | 1.47 [1.19, 1.83] | 54% | 0.01 | [0.06](https://0.xxx/) | - |
| Yap DY 2012 | 1.50 [1.23, 1.84] | 51% | [0.03](https://0.xxx/) | [0.05](https://0.xxx/) | No |
| Mok CC 2014 | 1.57 [1.29, 1.92] | 36% | [0.11](https://0.xxx/) | 0.03 | No |
| Zavada J 2010 | 1.49 [1.19, 1.86] | 58% | 0.008 | [0.07](https://0.xxx/) | No |
| Chen W 2012 | 1.52 [1.21, 1.92] | 56% | 0.01 | 0.07 | No |
| Chen W 2010 | 1.49 [1.17, 1.89] | 58% | 0.007 | 0.07 | No |
| Zheng Z 2022 | 1.49 [1.15, 1.92] | 59% | 0.007 | 0.08 | No |
| Li X 2012 | 1.47 [1.17, 1.85] | 58% | 0.007 | 0.07 | No |
| Liu Z 2015 | 1.43 [1.12, 1.81] | 54% | 0.02 | 0.07 | No |
| Rovin BH 2021 | 1.43 [1.13, 1.81] | 54% | 0.02 | 0.07 | No |
| Rovin BH 2018 | 1.41 [1.13, 1.76] | 52% | 0.02 | 0.06 | No |
| Z. Haitao, 2006 | 1.46 [1.18, 1.81] | 57% | 0.01 | 0.06 | No |
| Bao H 2008 | 1.45 [1.18, 1.76] | 50% | 0.03 | 0.05 | No |

# **Supplementary Table S7.** **PRISMA checklist**

| **Section and Topic** | **Item #** | **Checklist item** | **Location where item is reported** |
| --- | --- | --- | --- |
| **TITLE** | | |  |
| Title | 1 | Identify the report as a systematic review. | Page1 |
| **ABSTRACT** | | |  |
| Abstract | 2 | See the PRISMA 2020 for Abstracts checklist. | Page1 |
| **INTRODUCTION** | | |  |
| Rationale | 3 | Describe the rationale for the review in the context of existing knowledge. | Page2 |
| Objectives | 4 | Provide an explicit statement of the objective(s) or question(s) the review addresses. | Page2 |
| **METHODS** | | |  |
| Eligibility criteria | 5 | Specify the inclusion and exclusion criteria for the review and how studies were grouped for the syntheses. | Page2 |
| Information sources | 6 | Specify all databases, registers, websites, organisations, reference lists and other sources searched or consulted to identify studies. Specify the date when each source was last searched or consulted. | Page2 |
| Search strategy | 7 | Present the full search strategies for all databases, registers and websites, including any filters and limits used. | Page2 |
| Selection process | 8 | Specify the methods used to decide whether a study met the inclusion criteria of the review, including how many reviewers screened each record and each report retrieved, whether they worked independently, and if applicable, details of automation tools used in the process. | Page3 |
| Data collection process | 9 | Specify the methods used to collect data from reports, including how many reviewers collected data from each report, whether they worked independently, any processes for obtaining or confirming data from study investigators, and if applicable, details of automation tools used in the process. | Page3 |
| Data items | 10a | List and define all outcomes for which data were sought. Specify whether all results that were compatible with each outcome domain in each study were sought (e.g. for all measures, time points, analyses), and if not, the methods used to decide which results to collect. | Page3 |
|  | 10b | List and define all other variables for which data were sought (e.g. participant and intervention characteristics, funding sources). Describe any assumptions made about any missing or unclear information. | Not applicable |
| Study risk of bias assessment | 11 | Specify the methods used to assess risk of bias in the included studies, including details of the tool(s) used, how many reviewers assessed each study and whether they worked independently, and if applicable, details of automation tools used in the process. | Page3 |
| Effect measures | 12 | Specify for each outcome the effect measure(s) (e.g. risk ratio, mean difference) used in the synthesis or presentation of results. | Page3 |
| Synthesis methods | 13a | Describe the processes used to decide which studies were eligible for each synthesis (e.g. tabulating the study intervention characteristics and comparing against the planned groups for each synthesis (item #5)). | Page3 |
|  | 13b | Describe any methods required to prepare the data for presentation or synthesis, such as handling of missing summary statistics, or data conversions. | Not applicable |
|  | 13c | Describe any methods used to tabulate or visually display results of individual studies and syntheses. | Page3 |
|  | 13d | Describe any methods used to synthesize results and provide a rationale for the choice(s). If meta-analysis was performed, describe the model(s), method(s) to identify the presence and extent of statistical heterogeneity, and software package(s) used. | Page3 |
|  | 13e | Describe any methods used to explore possible causes of heterogeneity among study results (e.g. subgroup analysis, meta-regression). | Page3 |
|  | 13f | Describe any sensitivity analyses conducted to assess robustness of the synthesized results. | Page3 |
| Reporting bias assessment | 14 | Describe any methods used to assess risk of bias due to missing results in a synthesis (arising from reporting biases). | Page3 |
| Certainty assessment | 15 | Describe any methods used to assess certainty (or confidence) in the body of evidence for an outcome. | Page3 |
| **RESULTS** | | |  |
| Study selection | 16a | Describe the results of the search and selection process, from the number of records identified in the search to the number of studies included in the review, ideally using a flow diagram. | Page3-4 |
|  | 16b | Cite studies that might appear to meet the inclusion criteria, but which were excluded, and explain why they were excluded. | Not applicable |
| Study characteristics | 17 | Cite each included study and present its characteristics. | Page3 |
| Risk of bias in studies | 18 | Present assessments of risk of bias for each included study. | Page5 |
| Results of individual studies | 19 | For all outcomes, present, for each study: (a) summary statistics for each group (where appropriate) and (b) an effect estimate and its precision (e.g. confidence/credible interval), ideally using structured tables or plots. | Page5 |
| Results of syntheses | 20a | For each synthesis, briefly summarise the characteristics and risk of bias among contributing studies. | Page5 |
|  | 20b | Present results of all statistical syntheses conducted. If meta-analysis was done, present for each the summary estimate and its precision (e.g. confidence/credible interval) and measures of statistical heterogeneity. If comparing groups, describe the direction of the effect. | Page5 |
|  | 20c | Present results of all investigations of possible causes of heterogeneity among study results. | Page5 |
|  | 20d | Present results of all sensitivity analyses conducted to assess the robustness of the synthesized results. | Page9 |
| Reporting biases | 21 | Present assessments of risk of bias due to missing results (arising from reporting biases) for each synthesis assessed. | Page9 |
| Certainty of evidence | 22 | Present assessments of certainty (or confidence) in the body of evidence for each outcome assessed. | Page9 |
| **DISCUSSION** | | |  |
| Discussion | 23a | Provide a general interpretation of the results in the context of other evidence. | Page12-13 |
|  | 23b | Discuss any limitations of the evidence included in the review. | Page13-14 |
|  | 23c | Discuss any limitations of the review processes used. | Page13-14 |
|  | 23d | Discuss implications of the results for practice, policy, and future research. | Page15 |
| **OTHER INFORMATION** | | |  |
| Registration and protocol | 24a | Provide registration information for the review, including register name and registration number, or state that the review was not registered. | Page2 |
|  | 24b | Indicate where the review protocol can be accessed, or state that a protocol was not prepared. | Not applicable |
|  | 24c | Describe and explain any amendments to information provided at registration or in the protocol. | Not applicable |
| Support | 25 | Describe sources of financial or non-financial support for the review, and the role of the funders or sponsors in the review. | Page14 |
| Competing interests | 26 | Declare any competing interests of review authors. | Page14 |
| Availability of data, code and other materials | 27 | Report which of the following are publicly available and where they can be found: template data collection forms; data extracted from included studies; data used for all analyses; analytic code; any other materials used in the review. | Not applicable |

*From:*  Page MJ, McKenzie JE, Bossuyt PM, Boutron I, Hoffmann TC, Mulrow CD, et al. The PRISMA 2020 statement: an updated guideline for reporting systematic reviews. BMJ 2021;372:n71. doi: 10.1136/bmj.n71
